# A quantum microtubule substrate of consciousness is experimentally supported and solves the binding and epiphenomenalism problems

**DOI:** 10.1093/nc/niaf011

**Published:** 2025-05-06

**Authors:** Michael C Wiest

**Affiliations:** Department of Neuroscience, Wellesley College, 106 Central St., Wellesley, MA, United States

**Keywords:** microtubules, anesthesia, quantum consciousness, Orch OR, orchestrated objective reduction, hard problem, binding problem, epiphenomenalism, panpsychism

## Abstract

Recent experimental evidence, briefly reviewed here, points to intraneuronal microtubules as a functional target of inhalational anesthetics. This finding is consistent with the general hypothesis that the biophysical substrate of consciousness is a collective quantum state of microtubules and is specifically predicted by the Orchestrated Objective Reduction theory of Penrose and Hameroff. I also review experimental evidence that functionally relevant quantum effects occur in microtubules at room temperature, and direct physical evidence of a macroscopic quantum entangled state in the living human brain that is correlated with the conscious state and working memory performance. Having established the physical and biological plausibility of quantum microtubule states related to consciousness, I turn to consider potential practical advantages of a quantum brain and enormous theoretical advantages of a quantum consciousness model. In particular, I explain how the quantum model makes panprotopsychism a viable solution to physicalism’s hard problem by solving the phenomenal binding or combination problem. Postulating a quantum physical substrate of consciousness solves the binding problem in principle but appears to leave us with an epiphenomenalism problem, meaning that consciousness seems to have no causal power to confer a fitness advantage, so its evolution remains as an inexplicable mystery. I propose that, contrary to a certain (zombie) intuition, the quantum approach can also solve this problem in a nontrivial way. The Orchestrated Objective Reduction (Orch OR) theory of Penrose and Hameroff embodies these advantages of a quantum model and also accounts for nonalgorithmic human understanding and the psychological arrow of time.

## Anesthesia and consciousness

Inhalational anesthetics bind promiscuously at hydrophobic pockets in a variety of proteins in the brain and spinal cord. They are currently believed to cause unconsciousness by acting on some combination of ion channels and receptors, synaptic proteins and gap junctions, mitochondria, and cytoskeletal proteins including microtubules (MTs; Hemmings et al. 2019, Kelz and Mashour 2019, Mashour 2024).

**Figure 1.**
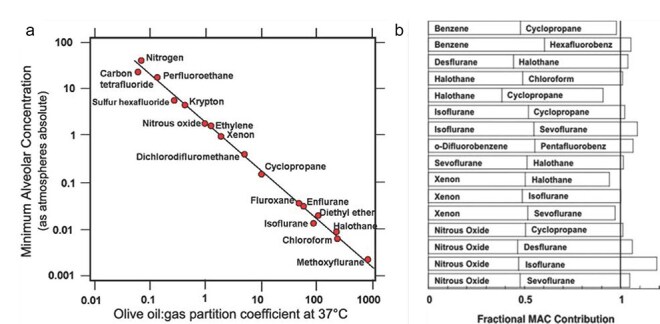
Anesthetic properties suggesting a common “unitary” molecular target. (a) The Meyer–Overton correlation for inhalational anesthetics. ‌The effective dose (vertical axis) is predicted by solubility in olive oil (horizontal axis), suggesting a weak physical interaction at an evolutionarily conserved lipophilic target, rather than chemical lock-and-key binding. (b) Additivity of effective doses (of “MACs”). One-half the effective dose of one anesthetic plus one-half the effective dose of another anesthetic equals one effective dose, even when the two anesthetics have very different effects on a particular ion channel, arguing against ion channels as the primary functional target of inhalational anesthetics. Figure reproduced from Eger et al. (2008) with permission from Wolters Kluwer Health, Inc. The Creative Commons license does not apply to this content.

On the other hand, multiple striking empirical facts appear to suggest that these diverse anesthetic compounds act primarily on a single highly conserved molecular target protein to “selectively” abolish consciousness. First, there is the venerable Meyer–Overton correlation (Katz 1994) between anesthetic potency and solubility in olive oil over several orders of magnitude (Fig. 1a). It suggests that anesthetics interact via weak physical interactions such as van der Waals forces rather than ionic binding. Moreover, if the anesthetics target multiple molecular targets, this result seems to imply that all the targets in diverse proteins share highly similar binding properties. A unitary molecular target might be more plausible than a varying combination of ion channels and other targets.

Second, the effective dose for a given anesthetic varies little across diverse species (Eger et al. 2008), despite wide variability in the profiles of different ion channels in each animal. A third remarkable property of these compounds is the approximately linear additivity of their effects, despite variable effects of each anesthetic on specific ion channel targets (Fig. 1b). For example, isoflurane activates Gamma-aminobutyric acid (GABA) receptors relatively strongly, while cyclopropane has only a small effect—but nevertheless half of an effective dose of isoflurane plus half the effective dose of cyclopropane results in a full effective dose. Figure 1b shows several such combinations chosen for their differential effects on a particular candidate ion channel. Examples like these appear to show that no single ion channel can account for the unconsciousness caused by inhalational anesthetics. Importantly, a systematic analysis of available evidence also appeared to show that no combination of ion channel targets can account for the pattern of empirical results either (Eger et al. 2008), leaving those authors perplexed. Since then the field has tended to assume that anesthesia is mediated by a combination of ion channels and other targets (Hemmings et al. 2019, Mashour 2024), but again, this approach fails to account for the Meyer–Overton correlation and other remarkable facts we noted earlier.

However, these analyses did not rule out MTs as the primary molecular mediator of inhalational anesthesia. Indeed, volatile anesthetics bind to MTs (Pan et al. 2008) resulting in altered gene expression (Futterer et al. 2004, Kalenka et al. 2007), and a behavioral experiment demonstrated that an anthracene-based anesthetic reversibly immobilized tadpoles by acting on their MTs (Emerson et al. 2013). Similarly, a clinical study of anesthetic usage by human surgery patients given MT-stabilizing chemotherapy (which generally penetrates poorly into the brain) compared to control subjects found a slight anesthetic resistance in the patients given the MT-binding drug (Linganna et al. 2015). Most recently, my lab reported that rats administered with a brain-penetrant MT-binding drug took significantly longer to fall unconscious under the volatile anesthetic isoflurane (Khan et al. 2024), suggesting that isoflurane causes unconsciousness at least in part by binding to MTs. The effect size was “large” as assessed by a Cohen’s *d* value of 1.9.

Remarkably, a detailed quantum chemical modeling study found that the potencies of several volatile anesthetics were predicted by their binding affinity to delocalized electron sites within the tubulin subunits that make up MTs (Craddock et al. 2015, 2017). These theoretical results essentially reproduce the Meyer–Overton correlation by assuming that anesthesia is primarily mediated by MTs. This cannot be said for any other candidate molecular target. Thus, MTs could be the primary molecular target that mediates the unconsciousness caused by inhalational anesthetics.

This is not to say that no other molecular targets contribute to anesthesia. For example, binding to GABA receptors does appear to contribute to unconsciousness caused by isoflurane because mice with nonfunctional GABARs are somewhat resistant to isoflurane (Sonner et al. 2007). To a first approximation, if binding to MTs can explain the Meyer–Overton correlation between anesthetic potency and binding at a specific lipophilic site (Craddock et al. 2017), then contributions from other mechanisms would represent the deviations from the linear relation visible in Fig. 1a.

Taking for granted that MTs are one of the functional targets of volatile anesthetics, we can consider how this might be accounted for by contemporary classical neuroscientific models of consciousness. In these models (Dehaene et al. 1998, Sergent and Dehaene 2004, Tsuchiya et al. 2015, Tononi et al. 2016, Mashour et al. 2020, Albantakis et al. 2023), the substrate of consciousness is understood to be a pattern of membrane electrical activity across a population of distributed neurons. Although spiking activity is somewhat reduced under anesthesia (Garcia et al. 2010), unconsciousness is understood to be the result of functional disconnection among brain regions rather than reduced brain activity *per se* (Hemmings et al. 2019, Mashour 2024). Under this picture, the most natural way to understand how perturbing MTs would contribute to unconsciousness would be via an effect on synaptic transmission. (However, as discussed earlier, such a picture still fails to account for the multiple facts suggesting a unitary molecular target.) Since MTs form the tracks for intracellular transport of synaptic proteins and transmitters, disrupting them might conceivably contribute to reducing synaptic transmission, and thus indirectly reduce firing and functional connectivity. In fact the anesthetic isoflurane does inhibit synaptic transmission, but the mechanisms by which it does so do not appear to involve MTs (Herring et al. 2009, Zimin et al. 2018), so this possibility does not seem to be supported at present. A more radical suggestion would be to consider a classical cellular-automaton process on MTs as the substrate of consciousness, but this possibility does not appear particularly plausible or attractive given the dramatic advantages of a quantum model, as discussed in later sections.

## MTs are biological integrators well suited for two-way communication with membrane electrical activity

So let us consider the quantum hypothesis: that anesthetics cause unconsciousness by disrupting a delicate entangled collective quantum state of many neural MTs that constitutes the direct substrate of consciousness. The susceptibility of the coherent quantum state to disruption by relatively weak binding explains why the anesthetic effect is specific to consciousness (at moderate doses) despite the promiscuous binding of anesthetics to many targets. Anesthetic quantum binding results in randomization of quantum processes in target proteins, disrupting highly orchestrated and entangled quantum activities. Binding to all the other nonorchestrated proteins has no effect because those quantum processes are already incoherent. Beyond that, neural MTs are identified as the substrate of the quantum conscious process by the Orch OR theory (Penrose and Hameroff 1995, Hameroff and Penrose 1996, 2014, Hameroff 2021) because they appear to offer an ideal candidate medium for intracellular integration and adaptive responding.

Why MTs? MTs are well situated in neurons to integrate electrical activity on the neural membrane (e.g. via calcium influx during neural activity) and in turn modulate neural membrane voltages and spiking activity (e.g. by modulating synaptic release probabilities, which are known to be stochastic, “noisy,” or “unreliable”). They are evolutionarily plausible candidates because they are present in all animal cells and perform numerous cellular functions related to the integration of environmental signals and coordinating movement. They conduct the beautiful dance of chromosomes during cell division, they are responsible for the movement of cilia and flagella (along with other proteins), and they can even function to detect and respond to the direction and orientation of electromagnetic signals (Albrecht-Buehler 1991, 1992, 1994, 1995)—like a cellular eye and brain! This allows us to understand our consciousness as an elaboration of more primitive processes that already existed in biology. Remarkably, the same anesthetic vapors that make us unconscious also reversibly slow or halt motility in single-celled organisms and plants (Kelz and Mashour 2019, Yokawa et al. 2019). This led Claude Bernard to assert: “What is alive must sense and can be anesthetized. The rest is dead.” Postulating a quantum state of MTs as the substrate of consciousness that is sensitive to volatile anesthetics explains why those anesthetics also work on single cells with no neurons.

The idea that MTs in a living brain could support stable macroscopic quantum effects has generally been considered extremely implausible since Tegmark’s influential estimate of decoherence times in the brain (Tegmark 2000). However, as pointed out by Hagan et al. (2002) shortly thereafter, Tegmark assumed tubulin proteins (MT subunits) would have to form superpositions of distinct positions separated by an unrealistically large distance. Moreover, Tegmark assumed thermal equilibrium in his calculations, which is equivalent to death, and thus not appropriate for describing living matter. In fact, earlier biophysical models (Frohlich 1968, 1977, Wu and Austin 1981) had demonstrated how systems of electric dipoles (like tubulin subunits of MTs) can “condense” into stable coherent states at high temperatures if a steady supply of metabolic energy is pumped through the system. In such a steady state, the system is explicitly far from equilibrium, in contradiction with Tegmark’s analysis. Where Tegmark’s analysis concluded the brain is too hot to support stable quantum functions, a later analysis in terms of a nonequilibrium Frohlich-type model concluded the brain is too cold, at body temperature, to support functional quantum states (Reimers et al. 2009). However, this later study modeled one-dimensional polymers rather than tubular MTs (Salari et al. 2011).

In any case, experiments have now demonstrated nontrivial quantum effects in MTs at room temperature. This includes direct evidence of quantum super radiance from MTs at room temperature, which was enhanced as they were joined into larger structures (Babcock et al. 2024). Similarly, Anirban Bandyopadhyay and colleagues stimulated MT resonances in cultured neurons and observed the MT resonance state “spanning across multiple neurons and controlling membrane voltage” (Saxena et al. 2020, Singh et al. 2021a, b). These experiments strongly support the physical plausibility of the quantum MT consciousness hypothesis. Moreover, quantum optical effects in MTs were shown to be dampened by inhalational anesthetics (Kalra et al. 2023), supporting the view of anesthesia I outlined in the previous section.

## Experimental evidence for, and potential advantages of, a quantum conscious process in the human brain

In a recent series of experiments, Kerskens and Pérez (2022) and Pérez et al. (2023) used a novel quantum entanglement-detection method applied to conscious humans in a magnetic resonance imaging (MRI) scanner. They reported strong evidence for an entangled brain state related to consciousness and working memory performance. They used an unconventional MRI protocol designed to isolate signals from entangled states and observed an MRI signal that mimicked heartbeat-evoked potentials recorded with electromyogram. The authors argued the observed signal implied the existence of an entangled brain state that was capable of coupling with the nuclear spins in water molecules that were entrained by the MRI machine. Because the fidelity of the putative spin-entanglement signal correlated with short-term memory performance (Pérez et al. 2023) and the presence or absence of the conscious state itself in sleep vs. waking (Kerskens and Pérez 2022), the authors concluded that the quantum brain processes are likely an important part of our cognitive and conscious brain functions. Their interpretation in terms of entanglement has been challenged (Warren 2023), but that author offered no alternative classical account of the signal observed by Kerskens and Pérez. Interestingly, anesthesia research has also implicated nuclear spin in the mechanism of anesthesia (Li et al. 2018). This result is baffling on the assumption that anesthetics work by standard chemical (i.e. electronic) binding at receptors, but understandable in terms of a quantum model of consciousness (Hameroff 2018).

Aside from this direct biophysical evidence, we also have a substantial body of behavioral evidence that human cognition is essentially quantum in nature (Wang et al. 2014, Pothos and Busemeyer 2022). The quantum probability formalism is distinct from classical Bayesian probability theory and produces behaviors (choices and judgments) that are suboptimal and irrational from the classical point of view. This would appear theoretically undesirable, but quantum formalism provides a unified description of actual human choice behaviors (Bordley 1998, Atmanspacher et al. 2004, Bruza et al. 2009, Conte et al. 2009b, a, Blutner et al. 2013, Pothos and Busemeyer 2013a, b; Wang et al. 2014, Bruza et al. 2015, Busemeyer et al. 2017, Zhu et al. 2020, Pothos and Busemeyer 2022) that are often accounted for by multiple *ad hoc* heuristics under the classical framework (Gigerenzer and Selten 2001; Lewis et al. 2014). Examples include the “conjunction fallacy,” in which people judge the probability of two events occurring together as more probable than one occurring alone (e.g. Linda is a feminist bank teller judged more probable than Linda is a bank teller), and violations of the “sure-thing principle,” such as a person willing to invest if the economy declines or improves—but declining to invest when the economy is uncertain. The quantum cognition framework has also generated novel, nontrivial quantitative predictions borne out by robust experimental evidence, particularly with regard to question order effects in which people give different answers depending on the order in which pairs of questions are posed (Wang et al. 2014, Pothos and Busemeyer 2022). It is conceivable that this body of data might be explained by purely classical neural network mechanisms (Busemeyer et al. 2017, Costello and Watts 2018, Costello et al. 2018), but the plausibility and generality of such schemes are questionable.

### The quantum advantage

Identifying the unity of conscious states with the unity of a quantum brain state (as discussed in later sections) also conceptually solves the problem of explaining how conscious brain states could have evolved. A brain state that is objectively unified in this way accounts for physical advantages conferred by the quantum process—quantum computational advantages including quantum associative memory advantages (Ventura and Martinez 2000, Schuld and Petruccione 2018)—while at the same time accounting for how that complex physical state distributed across the cortex could correspond to a unified experience.

In particular, the potential memory advantage of a quantum brain should not be overlooked. A currently fashionable opinion asserts that the promise of quantum computation is mostly hype because there are few useful quantum algorithms. Shors’ factorization algorithm is the most famous quantum algorithm because it offers exponential speedup relative to the classical analog, but who needs to factor large numbers unless you are trying to rob a bank? But speed is not the only potential advantage of quantum computing.

Combining Grover’s more modest search algorithm with a neural network architecture led to an exponential increase in memory capacity as compared to classical neural network models of associative memory (Ventura and Martinez 2000). It is common to estimate the brain’s capacity by calculating the number of possible combinations of activity in different individual neurons. For example, in a model with four neurons in a spiking or nonspiking state of activity, we would calculate 2^4^ = 16 possible memory states. Under this viewpoint, the memory capacity increases exponentially with the number of neurons, reaching fantastical values. But actual classical neural network models of associative memory like the Hopfield model have capacities that grow only linearly with the number of neural units. In a network of 100 neurons, you might only be able to reliably store 15 or 20 memory patterns. Not 2^100^.

This is a significant issue for neuroscience because humans have immense memory capacity (Standing 1973, Brady et al. 2008) that may not be accounted for by current models. Consistent with the Orch OR account, the cytoskeleton does play a major role in memory (Craddock et al. 2012). These considerations are not intended as conclusive evidence that human cognition necessarily relies on quantum probability or quantum memory. Rather, they represent circumstantial evidence consistent with the quantum view I am defending, and a plausible evolutionary advantage of a quantum brain process.

More broadly, having unified quantum states to compute with means an organism can make decisions based on the current whole configuration of sensory information and memories: the whole situation. This is exactly what conscious experiences enable.

Moreover, the most fundamental formulation of quantum dynamics involves a “path integral” over all conceivable ways the system could evolve. The quantum path integral combines all the possibilities in such a way that the actual evolution of the system minimizes the physical “action.” For our present purposes, we need only appreciate that this means the universal dynamic can be understood as an optimization process. It is as if Nature considers all possible paths forward, and decides on an optimal one. If organisms have evolved a way to map their own personal problems onto this natural optimization dynamic, it could function as a powerful tool for generating optimal adaptive behavior.

This vague conjecture is realized in an impressive concrete form in a fascinating quantum model of visual pursuit—how our brain moves our eyes to follow a moving visual target (Behera et al. 2005). The quantum model dramatically outperforms comparable classical algorithms. Moreover, unlike the classical algorithms, the quantum model predicts occasionally discontinuous jumps (saccades) in eye position, as is observed experimentally.

Similarly, human hand and arm movements are controlled near-optimally. In particular, trial-to-trial fluctuations in real movements tend to vary flexibly along task-irrelevant dimensions, which can change quickly as one adopts new goals (Todorov and Jordan 2002, Todorov 2009). Such fast, adaptive adjustments to ongoing behaviors might be naturally accounted for by a generalization of the quantum saccade model, which models optimal control of a simpler motor behavior. Friston and colleagues have developed an impressive general “active inference” formalism for path-integral-style optimization of behavior and learning (Pezzulo et al. 2024), but it is missing a realistic physical implementation in the brain. I suggest that the Orch OR theory provides the missing substrate for implementing active inference: the quantum dynamics of the collective MT state in the brain.

*

We have seen that anesthetic mechanism research points to MTs as a likely substrate of consciousness. We have discussed experimental observation of quantum effects in MTs at room temperature, and MT resonances spanning multiple living neurons and interacting with membrane voltage. We have noted significant potential behavioral advantages of a quantum brain process. And we have direct MRI evidence of a macroscopic quantum state related to consciousness in the human brain. Let us now turn to explore the theoretical motivation for considering a quantum model of consciousness in the first place.

In their 1996 paper describing the Orch OR proposal, Penrose and Hameroff listed “problematic feature[s] of consciousness” and their possible solutions under the quantum model. The first listed problem was the “unitary sense: the ‘binding problem.’” The proffered quantum solution was “(1) Non-local quantum coherence; indivisible macroscopic quantum state (e.g. Bose-Einstein condensate). (2) Instantaneous *self*-collapse of superposed stated (Orch OR).” This problem and solution pair was offered in Table 1 of (Hameroff and Penrose 1996) but not discussed in the text. Penrose and Hameroff have repeatedly claimed that Orch OR solves the binding problem (BP), without much explanation (Hameroff and Penrose 2014). What did they mean?

I submit that what Penrose and Hameroff meant by the “binding problem” is referring to what has become known as the “combination problem of panpsychism” by philosophers (Seager 1995, Goff 2009). Furthermore, the proffered quantum solution is what Chalmers identified as the most promising candidate solution to the combination problem (CP) in his paper on panpsychism and panprotopsychism (to be defined later) (Chalmers 2013)—but he despaired of finding a viable neural basis for the idea.

In defending Orch OR over the years, Penrose and Hameroff naturally focused on their specific contributions and expertise—the biology of MTs, the physics of wave function collapse, and the highly technical and subtle Gödel argument (to be briefly reviewed later). This is unfortunate because the motivation to solve the BP, to account for the “unitary sense,” aka the “unity of consciousness,” is the primary motivation for the quantum approach, and it derives from a more straightforward argument.

To anticipate my upcoming argument, below I will review the problem of accounting for phenomenal properties, known as the hard problem (HP), and outline the physicalist and panpsychist approaches to solving it, before settling on panprotopsychism as a potential solution to the HP. But panprotopsychism comes with its own very difficult problem, the CP (which, as noted later, is also a problem for physicalism). I will review the CP and describe how it is related to the phenomenal BP that is more familiar to neuroscientists. Failing to solve the CP will be considered “fatal” to any theory. Thus far the discussion makes no reference to the quantum hypothesis. The quantum hypothesis is introduced as a solution to the CP to make panprotopsychism viable as a solution to the HP. We will then move on to another fundamental problem that I argue can be solved by the quantum approach, which I will refer to as the epiphenomenalism problem (EP).

## Physicalism, the HP, and panprotopsychism

The most important and contentious issue in the contemporary study of consciousness is the question of whether it is necessary to introduce a new fundamental property to the list of fundamental physical properties described by physics, to account for consciousness, aka “phenomenal properties.” The HP (Chalmers 1997) refers to the apparent impossibility of deducing the presence of conscious experiences from the physical description of a system like a brain or anything else.

Physicalism (or materialism) is “the doctrine that the physical facts about the world exhaust all the facts, in that every positive fact is entailed by the physical facts” (Chalmers 1997). Of course, this definition is empty until we define “physical.” Following Chalmers, I take this term to refer to:

… the fundamental properties that are invoked by a completed theory of physics. Perhaps these will include mass, charge, spatio-temporal position…. High-level properties such as juiciness, lumpiness, giraffehood, and the like are excluded, even though there is a sense in which these properties are physical. In what follows, talk of physical properties is implicitly restricted to the class of fundamental properties unless otherwise indicated. I will sometimes speak of “microphysical” … properties to be explicit (Chalmers 1997).

Chalmers (2013) reports that “it is most common to restrict physical properties in this sense.” This restricted sense is termed the “narrowly physical” by him. This is just maintaining the fundamental distinction between “extension” and “thought” introduced into modern Western philosophy by Descartes and perfected by Spinoza. However, it is important to note that contemporary philosophers have proposed varied deviations from this traditional use of the term, in which the term “physical” allows for fundamental mental properties such as phenomenal consciousness (Strawson 2006, Stoljar 2024). Here I will use in term physical and physicalism in the “narrow” sense, which again simply means that they exclude fundamental phenomenal properties or fundamental mental properties of any kind. Under this definition, panpsychism and panprotopsychism, to be discussed later, are mutually exclusive with physicalism.

The HP is physicalism’s characteristic problem (Chalmers 1997). The problem is that it appears evident that no combination of physical quantities amounts to or implies a conscious experience. It is a contemporary crystallization of the older “mind-body problem” and “explanatory gap”—why should there be any experience at all associated with a physical system?—whether it is a brain or a brick. Diverse contemporary thinkers have used diverse arguments to compellingly make the case that this HP is insoluble for physicalism (Jackson 1982, 1986, Chalmers 1997, Griffin 1998, Rosenberg 2004, Tononi and Koch 2015, Koch 2019, Seager et al. 2022, Stoljar 2024). Alternative viewpoints that do not recognize the HP as a valid problem are reviewed in Graziano (2024).

### Panprotopsychism solves the HP in principle

The term panpsychism refers to the doctrine that all physical reality is somehow permeated with mind. If the mental property is phenomenal consciousness, we have panpsychism proper, and if the fundamental mental property is not itself conscious, but has the potential to become conscious in the appropriate context, we call the theory panprotopsychism (Seager 1995, Chalmers 2013, Seager et al. 2022). For concreteness, I will restrict myself to panprotopsychism going forward, which “is the view that fundamental physical entities are proto-conscious” (Chalmers 2013).

Such a postulate sounds like a radical step, perhaps even like “cheating,” but remember we have added new postulates to physics regularly to account for newly discovered fundamental properties like electric charge, quantum spin, quark “color charges,” and others. It is not cheating. In 1997, Chalmers wrote “to bring consciousness within the scope of a fundamental theory, we need to introduce new fundamental properties and laws … here the fundamental laws will be *psychophysical* laws, specifying how phenomenal (or protophenomenal) properties depend on physical properties. These laws will not interfere with physical laws… Instead, they will be *supervenience* laws, telling us how experience arises from physical processes” (Chalmers 1997).

Across the landscape of modern panpsychist theories, the most important landmarks in our context are the early twentieth-century theories of Whitehead and Russell. Seager, Goff, and Allen-Hermanson judge that “the most significant development and defense of a panpsychist philosophy in the twentieth century was undoubtedly that of Alfred North Whitehead” (Seager et al. 2022). Whitehead’s metaphysic (Whitehead 1933) falls under the panprotopsychist type of panpsychism and contains the most sophisticated and developed philosophical account of the relation between unconscious and conscious mental states to date. His ontology is also relevant in our context because Penrose and Hameroff endorse it as the appropriate metaphysical framework for describing Orch OR (Hameroff and Penrose 2014). On the other hand, many recent panpsychist thinkers have developed versions of Bertrand Russell’s “neutral monism,” which also involves a “psycho-cerebral parallelism” (Russell 1929, p. 361) and may be considered a form of panpsychism (Seager et al. 2022). Russell acknowledged Whitehead as having made an “immense contribution” and presented his own theory as a “less revolutionary” and “somewhat simpler” scheme (Russell 1929, p. 132). The contemporary varieties include the panprotopsychist Orch OR theory. The Orch OR theory is panprotopsychist in that objective reduction (OR) events, aka wave function collapse events, are equated with “microphysical” processes that carry “microphenomenal” properties, which are “orchestrated” in the brain to create integrated “macrophenomenal” properties like our moments of complex conscious experience.

The panprotopsychist postulate inherent in Orch OR eliminates the HP and leaves us with the easier problems of empirically determining the psychophysical bridging laws. But panprotopsychism also faces a very difficult—potentially fatal—problem.

## The CP, the unity of consciousness, and the BP

“The combination problem for panprotopsychism is: how can protophenomenal properties combine to yield macrophenomenal properties?” (Chalmers 2017). Here protophenomenal properties are the individually unconscious mental properties we just postulated for our fundamental physical entities, and the macrophenomenal properties refer to large-scale complex moments of consciousness such as we experience.

**Figure 2.**
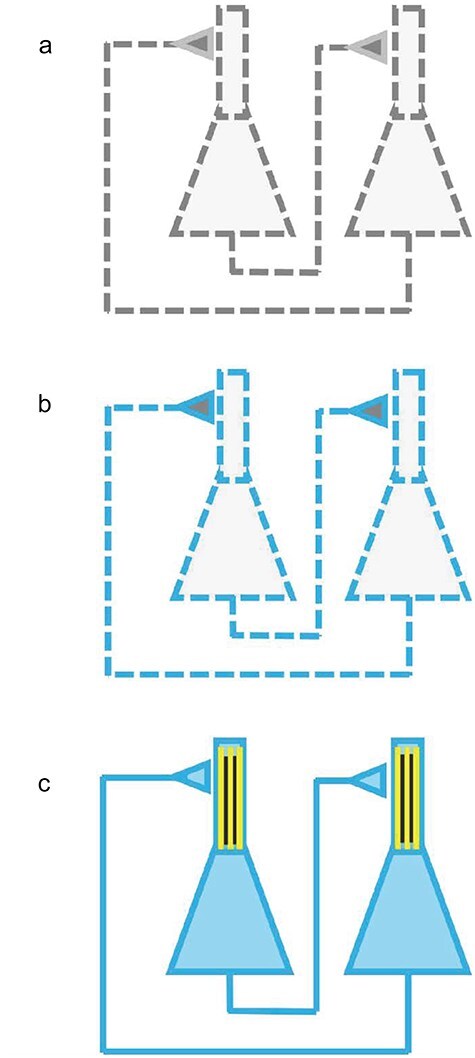
Solving the hard problem and the combination/binding problem. (a) A classical physicalist model contains no conscious properties (*grey color*) and no objective wholes: the system of neurons is completely reducible to neighbor interactions of local parts (represented by *dashed lines*) with no phenomenal properties (*grey color*). (b) A classical panpsychist model has conscious properties (*blue color*) but no objective wholes (separate *dashes*). (c)‌ The quantum panpsychist model contains causally efficacious objective wholes (*solid lines*) that correspond to unified conscious moments (*blue color*). *Vertical yellow lines* represent MTs in the dendrites, responsible for the unified conscious state.

Although this CP is traditionally discussed in the context of panpsychism and panprotopsychism (Seager 1995, Goff 2009, Chalmers 2017), it is critical to appreciate that “of course physicalism is faced with its own version of the combination problem: how do microphysical entities and properties come together to yield subjects, qualities, and so on? This challenge is presumably at least as hard as the challenge to panpsychism, as the resources available to the physicalist are a subset of those available to the panpsychist” (Chalmers 2017). Chalmers’ point is illustrated metaphorically in Fig. 2a and b. Figure 2b shows the CP for the panpsychist: how do the micro-mental properties (the dashes represent micro-entities like electrons that make up neurons, and their color represents their conscious or protoconscious property) combine to form a large-scale experience? Figure 2a illustrates that the problem for the physicalist is much worse: they do not have a mental property to begin with, so attempting to combine nonexistent microphenomenal properties (nonexistent color in the figure) to obtain conscious states, is like boiling sand hoping to get cooked rice. These metaphors of futility are ways of expressing the HP of physicalism. To solve this HP, we abandoned physicalism for panprotopsychism. Now we must grapple with the CP.

The proposal I am advancing is what Chalmers has called “quantum holism” (Chalmers 2017):

This view starts from the insight that on most common understandings of quantum mechanics, the fundamental entities need not be localized entities such as particles. Multiple particles can get entangled with each other, and when this happens it is the whole entangled system that is treated as fundamental and that has fundamental quantum-mechanical properties … ascribed to it. A panpsychist might speculate that such an entangled system, perhaps at the level of the brain or one of its subsystems, has microphenomenal properties. On the quantum holism version of … panpsychism, macrosubjects such as ourselves are identical to these fundamental holistic entities, and our macrophenomenal properties are identical to its microphenomenal properties.

Orch OR is the specific quantum panprotopsychist theory I am defending in this paper.

Chalmers continues: “This view has more attractions than the earlier views, but there are also worries. Some worries are empirical: it does not seem that there is the sort of stable brain-level entanglement that would be needed for this view.” This is the objection we refuted in the first part of the present study. Of the other potential objections to quantum panpsychism that Chalmers reviews, some are specific to the quantum hypothesis and some are not. He notes that a fundamental theory must ultimately address “all” the multiple aspects of the CP that have been identified in the literature. I am claiming that Orch OR will solve the CP completely. To begin to establish this thesis I will outline the most fundamental aspects of the problem identified by Chalmers’ analysis.

The “biggest worry” for quantum and nonquantum theories alike is called the “structural *mismatch problem*.” It refers to the apparent lack of correspondence—i.e. mismatch—between the detailed physical states of our brain and the details of our conscious experience. Spinoza, the first modern panpsychist, reasoned that: “In just the same way as thoughts and ideas of things are ordered and connected in the mind, so the affections of the body, or [in other words] images of things are ordered and connected in the body” (Spinoza 2005). Later philosophers, infected by the ontology of local deterministic pre-quantum physics, could not conceive how that thesis could possibly be true, so it grew to be perceived as an insoluble paradox or “antinomy.” The intuition that psycho-physical parallelism cannot be true, because our experiences do not appear to reveal the fundamental microconstituents they are supposedly made out of, is the mismatch problem. I will postpone addressing it until we have outlined a couple of other faces of the multi-headed CP.

“Some related worries are theoretical: On some interpretations of quantum mechanics the locus of entanglement is the whole universe…, on others there is no entanglement at all, and on still others there are regular collapses that tend to destroy this sort of entanglement.” I will lump this issue with Rosenberg’s boundary problem (Rosenberg 2004) and the problem of combining or separating subjects. The problem is this: if the physical state of the universe is described by a single entangled state, how do we account for separate individual “subjects” of experience? Let us call this the “separate subjects problem.”

In my view, the root from which all the faces of the CP are derived is what Chalmers calls the “unity problem”: “how do microexperiences come together to yield a unified consciousness?” The “unity of consciousness” refers to the fact that our every conscious moment is experienced as a whole, from a single point of view, even though it incorporates multiple different features and concepts (like colors, shapes, sounds, people, and objects). The unity I am referring to here is the unity of a conscious subject at a particular moment (the “specious present”), not the apparent unity of a subject across minutes or years. The unity of consciousness has been comprehensively defended by Timothy Bayne and others (Cleeremans et al. 2000, Bayne 2008, 2010). I take the unity of consciousness to be a real and characteristic feature of the phenomenon.

The issue of how to incorporate this unity of consciousness into a neural model is one way to express the so-called phenomenal BP (Treisman and Gelade 1980, Treisman 1998, Revonsuo and Newman 1999, Feldman 2013). In other words, since the color of an object is represented by neurons in one brain region (red, say), while its shape is represented in another (roundish), what is the physical basis of our holistic experience of the simultaneous combination (red-roundish apple)?

How is the BP related to the CP? The BP was historically defined in terms of brains, so it is a more restricted notion than the CP, which is a problem for a fundamental theory meant to apply to any arbitrary system. Thus, the BP may be understood as a special case of the CP. When Penrose and Hameroff proposed that their panprotopsychist quantum theory could solve the BP (Hameroff and Penrose 1996), the CP problem had only just been named (Seager 1995), so it is understandable that they did not use the newer more general term introduced in the philosophical literature.

In what follows I will first explain why a classical model must fail to solve the CP, and at the same time must also fail to account for the evolution of useful conscious states—we will call this the EP. Then I will explain how the quantum hypothesis addresses the unity, mismatch, and subject separation faces of the CP. Finally, we will see that because the quantum model can solve the CP, it can also solve the EP.

### Conscious causation

Let us first clarify how we are conceiving of causation under the panprotopsychist model. The form of panprotopsychism I am proposing does not allow for any causal interaction between the mental property and the physical properties of matter. Although we are following previous scientific practice in adding fundamental new properties to our physical theory, this is the first time we have added a property that is nonphysical in the sense that it cannot causally influence or be influenced by the physical.

“Conscious states” refers to brain states that happen to also be conscious. In other words, conscious states are physical states with physical properties like voltages, but they also have a mental property called “consciousness.” “Consciousness” refers to one particular property of that brain state, which includes qualia such as colors and feelings. Given the identification of moments of Orch OR with moments of conscious experience, we have four ways of conceiving the division of causal labor between the physical and mental properties.

The first three interpretations are consistent with the definition of physical I adopted earlier. They are the following:

1. causality is implemented by a neutral underlying reality with physical and mental properties (Spinoza/Whitehead/Russell),
2. causality is implemented by the physical properties and degrees of freedom alone (the “scientific default”), or
3. causality is implemented by protophenomenal properties alone (Rosenberg 2004).

My argument regarding epiphenomenalism below applies regardless of which of these three interpretations we adopt. However, because most scientists are used to equating physical properties with causal properties, my discussion below will refer to the physical properties as carrying the causal efficacy. This manner of speaking is consistent with the first two but not the third interpretation.

The fourth interpretation involves some kind of interaction between the mental property and physical properties of matter. An example would be a theory that proposes that “consciousness” selects or biases outcomes during wave function collapse. This class of theories has been analyzed extensively (Chalmers and McQueen, 2022). They are not ruled out because (quantum) physics is not causally closed. Another example of an interactionist theory would be a string theory whose “extra” dimensions are identified with protophenomenal dimensions and perceptual spaces. However, my purpose here is to show that we do not need to postulate (potentially problematic) interactions between the physical and mental properties to solve the EP, so I will restrict myself to the first three interpretations.

Aside from the issue of how mental properties relate causally to physical properties, discussed earlier, there is a related issue regarding whether consciousness arises too late to be involved in real-time control. This important issue is addressed from the Orch OR point of view in Hameroff (2012), but is beyond the scope of the present article.

### Classical models must fail to solve the CP and the EP

To proceed, I will take it for granted (i) that conscious states are causally efficacious as conscious states. That means the fact that those states are conscious has physical and behavioral consequences. It is just a restatement of the psychophysical parallelism we are postulating: there is something physically different about the macroconscious physical states. Although unconscious brain processes are capable of a variety of cognitive feats (Hassin 2013), the contemporary weight of evidence still favors the idea that conscious brain processes do confer specific computational and behavioral advantages (Baars 1988, Baumeister and Masicampo 2010, Baumeister et al. 2011, 2018, Mathieson 2024a, b).

I also accept the “unity thesis” discussed earlier: (ii) that conscious states are complex wholes—they combine a multiplicity into a unity. In other words, our conscious moments include multiple features like colors and shapes, but we experience those features as parts of a whole. Given these premises, rigorous reflection (Marshall 1989, Stapp 1995) shows that a system of classically interacting parts cannot provide a satisfactory substrate for causally effective spatially distributed wholes such as our conscious states. In other words, a classical model cannot solve the CP.

Why must this be true? Because the dynamics of any classical system are completely reducible to the local interactions among infinitesimal parts and their infinitesimally separated neighbors. If you doubt this, note that classical dynamics are always expressed in terms of differential equations. Special relativity makes explicit that all classical influences propagate locally with a finite speed. This means that any classical phenomenon that we describe in terms of a larger-scale complex object, such as a “tornado,” is completely reducible to the interactions of its local parts—the molecules of air. The “tornado” has no physical effects as a whole, so it has no objective existence as a whole. In a classical model, it only exists as a name that we give to a pattern of movement, for convenience. In principle, we need not ever refer to the “tornado” to completely specify the dynamics of the wind.

This is a problem for a theory of consciousness that has no complex wholes. We are referring to it as the BP, or CP, because of the failure to explain how the different features of our conscious experiences are “bound” into the single “combination” that we experience.

Indeed, the classical system will evolve in exactly the same way regardless of whether you give the pattern a name or just let your model follow its local dynamical rules. A well-known detailed analysis by Kim in terms of a concept of supervenience reaches the same conclusion: if a global consciousness is postulated to accompany physical states in a theory whose local dynamics completely specify a system’s evolution, then that consciousness must be epiphenomenal (Kim 1998). It cannot have any physical effects. This is the EP that I defined earlier. We see that it is closely related to the CP because large-scale wholes (“combinations” of lower-level entities) in the classical theory can always be eliminated from the description and are thus functionally epiphenomenal.

One might take the point of view that a Fourier description of a classical system in terms of large-scale waves could be just as fundamental as the position basis, like this:

“However, non-local degrees of freedom can be important even in classical physics, For instance, oscillations in a guitar string are local in Fourier space, not in real space, so in this case the “binding problem” can be solved by a simple change of variables. As Eddington remarked, when observing the ocean we perceive the moving waves as objects in their own right because they display a certain permanence, even though the water itself is only bobbing up and down. Similarly, thoughts are presumably highly non-local excitation patterns in the neural network of our brain, except of a non-linear and much more complex nature. In short, this author feels that there is no binding problem” (Tegmark 2000).

But the point is that in classical physics there is always the option of the local description. Therefore, any holistic entity in a classical model is epiphenomenal—the holistic entity can always be eliminated by a reduction to its smallest parts interacting locally. Therefore, a causally efficacious spatially distributed whole is ruled out in a classical physical model of the brain or anything else.

In the above-mentioned quote, Tegmark is making my case for me when he notes that “non-local degrees of freedom” like waves are observer-dependent, not objective, features, in a classical model: “when observing the ocean *we perceive* the waves as *objects in their own right*…” (emphasis added). They are not objects in their own right: they are only objects because they are being interpreted as such by a conscious observer. The exact same point applies to the “highly non-local excitation patterns in the neural network of our brain”—if you assume those excitation patterns are classical. Why? Again, because everything classical is reducible to local interactions.

The American psychologist William James recorded this insight 110 years before Tegmark wrote his paper, in his classic “Principles of Psychology”:

The ‘entire brain-process’ is not a physical fact at all. It is the appearance to an onlooking mind of a multitude of physical facts. ‘Entire brain’ is nothing but our name for the way in which a million molecules arranged in certain positions may affect our sense. On the principles of the corpuscular or mechanical philosophy, the only realities are the separate molecules, or at most the cells. Their aggregation into a ‘brain’ is a fiction of popular speech. Such *a fiction cannot serve as the objectively real counterpart to any psychic state whatever. Only a genuine physical fact can so serve*. (emphasis added; James 1890)

We always have the in-principle option to eliminate the larger scale objects from our description, without losing any predictive power of the theory, if the theory is classical. Thus, a classical theory offers no candidate physical substrates to account for the causal efficacy of our unified but complex states of consciousness. This failure of a classical model to provide for objective wholes (i.e. failure to solve the BP) is simultaneously a failure to account for the distinctive causal efficacy of holistic conscious brain states. The latter failure is the failure to solve what I am calling the EP. It means a classical model cannot account for the evolution of holistic conscious states such as we experience because every holistic property in a classical model may be eliminated in favor of a purely local description.

## The quantum model solves the BP/CP

### The unity problem

Multiple authors have argued that postulating a quantum substrate of consciousness solves the BP (or CP) (Marshall 1989, Stapp 1995, Hameroff and Penrose 1996, Chalmers 2013, 2017, Hameroff et al. 2014, Arkhipov 2022, Neven et al. 2024). Why? Because an entangled quantum state is an objective whole that nevertheless contains multiple parts and properties. It solves the unity problem ontologically.

This holism is genuine, unlike any imagined wholes in a classical model, because the quantum holism has physical consequences. In this sense it is irreducible; it cannot be eliminated. It is the irreducible holism of quantum physics that is responsible for its most “magical” phenomena.

Perhaps the best-known example is the so-called nonlocal correlations that occur when entangled particles are measured far from each other by independent observers. The particles coordinate in a way that cannot be accounted for in terms of signals propagating locally between them, as proven by Bell and confirmed experimentally multiple times at distances of kilometers (Miller 2016, Kaiser 2020). Einstein called it “spooky action-at-distance.” But there are other classically impossible quantum phenomena of cooperativity. They include superconductivity, in which electrons coordinate to pass between the nuclei of a superconducting material as current with zero electrical resistance, like ghosts passing through a wall. I have to also mention quantum computation algorithms that exploit the holistic nature of quantum states to achieve classically impossible computational feats.

The point of these examples is to establish that the holistic nature I am attributing to quantum states is not an optional characterization—it has experimentally verifiable physical consequences. The holism is mandatory and irreducible, not a poetic figure of speech. Bell proved that no local theory can account for the predictions of quantum mechanics—and the predictions of quantum mechanics are borne out by solid experiments over decades (Miller 2016, Kaiser 2020). Thus, the holistic, or nonlocal, character of quantum states is an irreducible objective property—there is no frame of reference or alternate description that eliminates it.

### The mismatch problem

This objective holistic property of quantum states is “realized,” or actualized, during OR, and is a natural mirror to the irreducible unity of our conscious experiences. When we recognize our unified conscious experience as a property of an objectively unified physical state, we can understand unity as the organic psychophysical bridging principle between matter and mind. This is the natural psychophysical bridging principle that David Chalmers was seeking in his seminal book *The Conscious Mind* (Chalmers 1997). The structure of entanglement throughout the system is a well-defined quantifiable physical property that is predicted under this hypothesis to correspond to the unified contents of consciousness being experienced by that system. Under the Orch OR model, the “match” must be sought in the correspondence between collective vibrational states of MTs and our moment to moment experience. The reason this predicted correspondence is plausible is that the MTs “feel” the detailed state of the distributed spatiotemporal pattern of electrical activity among populations of neurons via calcium influx that mirrors neural activity and modulates MTs and MT-associated proteins. This is the proffered solution to the mismatch problem, in outline. It only makes sense because quantum dynamics of OR allow the entangled microstates to be fused into a unified but complex macrostate at the moment of “decision,” encompassing all the information in the physical representation that is “realized” in consciousness (Fig. 2c).

As neuroscientists we already believe something comparable: that there is a physical substrate of consciousness, and a “neural code” to be discovered. The neural code postulated and partially demonstrated experimentally is a familiar example of a form of psychophysical correspondence (now formulated in terms of the concept of “supervenience”) that most scientists are accustomed to believe, based on extensive imaging and causal evidence in humans and other animals. The quantum viewpoint just pushes this correspondence further, by pointing out that there must be a physical instantiation or “representation” of the “integration” of the contents of consciousness that we experience—a physical “image” of the connections or relations among things in Spinoza’s terminology—not just an aggregate of isolated elements of our experience.

We noted earlier that William James demands an “objectively real counterpart” to the mind, and “only a genuine physical fact can so serve.” Classical physics simply has no objectively real irreducible wholes that could “match” our complex moments of experience: classical physics cannot solve the mismatch problem.

In contrast, our quantum model contains a physical property that we can identify as essential for large-scale consciousness such as ours to emerge as a property of a physical system: it must be a physical state in which the features and variables that are reflected in the experience must be supported by physical variables that participate in a single collective entangled quantum state (Fig. 2c). In the quantum model, the holism is mandatory and irreducible.

In our quantum panprotopsychist picture, the kinds of conscious states we experience emerge when the molecules in our brain are appropriately configured, in appropriate environmental conditions, so that their individual elementary properties are “orchestrated” to merge into a collective quantum coherent state (in terms of the physical properties of the system), which is experienced as a unified conscious state (in terms of the corresponding mental property of the system).

Does this mean that every quantum coherent or entangled state incorporating multiple particles or variables is conscious? No. In fact, the quantum nonlocality that is the essence of quantum holism does not manifest physically until collapse of the wave function during OR events. The physical unity is not “realized” until that moment of collapse. Until then the quantum dynamics are perfectly local as in a classical theory. We therefore require OR for a consistent quantum theory of consciousness, as in Orch OR, in which protoconscious events are identified with wave function collapse rather than with any entangled state. This is when the quantum holism of the entangled state of potentialities is actualized into physical reality with physical consequences. The objective holism of the quantum collapse process gives the theory an unambiguous handle, or again a “bridge,” by which to relate the essential property of the mental (holism) to a physical property (holistic behavior of quantum collapse dynamics). That means the quantum theory of consciousness, although it is only in embryonic form right now, could develop to systematically relate physical states to conscious states in a natural way—that is what we might call “understanding consciousness.”

### The separate subjects problem

In addition, equating conscious moments with orchestrated collapse events provides a Whiteheadian solution to the problem of separate subjects that we identified earlier. In Whitehead’s scheme, every actual entity participates in determining every other actual entity, but with varying degrees of “relevance.” So while it may be true that, under a quantum model, every body is entangled with every other body in the universe: conscious bodies have “orchestrated” these entanglements to exclude the irrelevant ones. This is possible because entanglement affects quantum amplitudes, which can cancel each other out in the quantum path integral, unlike classical probabilities. In other words, conscious subjects are separate from each other and their environment because their brains “average out” the incoherent entanglements with the environment. The “decision” as to the precise separate individuality of each conscious moment is actualized, and embodied, by the Orch OR of the MT state in the brain. This is the proffered solution to the problem of separate subjects.

It must be understood that the reduction event does not eliminate all entanglement within the system or between the system and its environment. Before collapse, the quantum state encompasses a superposition of multiple “classical” outcomes. However, each classical outcome remains a quantum state with its own profile of internal coherence and entanglement. The classical outcomes are considered as such because they are eigenstates of some macroscopic observable like position. But every such eigenstate is a superposition when written in terms of another basis, which one can always do. Thus, the reduction event should be understood as discontinuously establishing a new pattern of entanglement, coherence, and ontological unity, rather than eliminating all quantum effects. The new unity of the “next” moment is established discontinuously in the collapse, but this constitutes the unified entangled state that evolves deterministically and locally until the next collapse.

Another misunderstanding to be avoided is the notion that any “measurement” or scientific “observation” is a quantum reduction event. For example, under Orch OR there is no reason to assume that measuring scalp potentials via electroencephalogram or blood dynamics with fMRI would interfere with conscious function.

With this understanding, we can in principle predict the fusion and fission of subjects, as in split-brain patients. We get a principled way to predict when subjects fuse or divide, in contrast to a classical model which has no objective irreducible wholes in the first place (Fig. 2). In typical nonbiological systems, although entanglement may be plentiful, reduction events will tend to be “random” and incoherent, resulting in meaningless protoexperiences.

To summarize, the quantum hypothesis makes the panprotopsychist solution to the HP coherent and viable. Such a solution is literally inconceivable for a classical theory. We can say the quantum approach, combined with a new panprotopsychist postulate, does solve the HP at a general conceptual level and allows us to envision a more detailed solution and understanding that might be developed in the future.

*

Hold on! If the quantum theory can just postulate its way out of the HP, why can’t a classical theory make the same move?

Again, because the classical physical theory contains nothing that can rationally “correspond” to, or “match,” the unity of the mental experience, so the incipient classical panpsychist theory is killed in its cradle by the BP/CP (see Fig. 2b, in which the separate dashes of blue color represent separate microconsciousnesses incapable of fusion into a macroconsciousness). That means that a classical theory of consciousness can only aspire to being a descriptive catalog of states of matter listed alongside their associated conscious experiences (in physical systems where those are independently known to occur). A classical theory is like an infinite list of arbitrary states of matter that are conscious, which is also powerless to predict whether configurations of matter not on the list will be conscious and what form that conscious experience might take. In an *ad hoc* theory like this, we have no way of systematically relating the classical physical states to the associated mental states.

Moreover, in a classical model, if we postulate that multiple distinct physical properties—like the firing rates or voltages of neurons in different parts of the brain at the same moment—are experienced together consciously, then this holistic experience cannot actually affect the animal’s behavior, so there is no way to understand how such experiences could have evolved. Instead they become *ad hoc* arbitrary postulates tacked onto physics: certain states of certain physical systems called brains are conscious, but the consciousness does not do anything and there is no way to account for why certain groupings of activities are conscious while others are not. We do not get any predictions about whether our attempts at artificial intelligence are conscious, or about when consciousness begins in humans, or whether insects or space aliens are conscious. This is an elaboration of my argument mentioned earlier, that a classical model must fail to solve the EP, which means a failure to account for the biological evolution of brain states that are useful because they are conscious.

## The quantum model solves the EP

Would the potential advantages of a quantum brain that we reviewed earlier be epiphenomenal? Of course not. They are potential advantages of a specific kind of physical system (i.e. an entangled quantum process), which by definition carries physical causal power. The EP refers to the evident fact that if the physical dynamics describing a system’s behavior fully determine its behavior, then any additional mental properties we postulate can have no effect on the system’s behavior. But then we cannot understand why our conscious states appear to offer “useful” information about the environment and good advice (e.g. “take your hand out of the fire!”). In other words, if our experiences can have no effects, how can they be useful? How could they have evolved?

An epiphenomenon is some property or feature of a system that has no functional effects on the operation of that system. If conscious physical states confer a fitness advantage, the property of consciousness itself could be said to not be responsible for the behavioral advantage; it would instead be the “physical” property of the conscious physical state that confers the advantage via “its” causal power.

**Figure 3.**
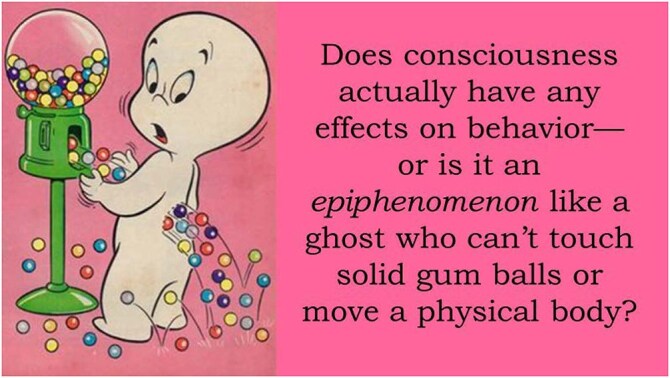
The solution to the epiphenomenalism problem is to recognize a *necessary relation* between the unity of consciousness and the objective efficacious unity of the quantum physical substrate. This relation accounts for behavioral advantages conferred on organisms able to implement quantum conscious processes. In contrast, a classical model cannot solve the epiphenomenalism problem because its large-scale wholes—like our conscious states—are *reducible* to locally interacting parts, and may thus be *eliminated* from the description without changing the model’s empirical predictions. This is a problem because the classical model cannot explain how our conscious states could have evolved and how they could be *useful*.

Instead of appealing to an interaction between consciousness and its physical basis to rescue the evolutionary relevance of consciousness, we have developed the idea that certain physical properties (holism) are “implied” by certain properties of conscious states (experienced unity) if those states are to be efficacious as conscious states (Fig. 3). In other words, we identified a natural objective bridging principle, rather than settling for an arbitrary inexplicable relation between the property of consciousness on the one hand and the physical substrate on the other.

Remember from our discussion of the BP and EP earlier, that even if a classical model postulates that such and such physical state is experienced consciously, the postulated conscious experience must be an epiphenomenal ghost that can never influence the behavior of the physical system, so the theory can never explain how conscious states could have evolved to confer a fitness advantage to the conscious organism. Why must it be epiphenomenal? Again, because it can be eliminated without changing the explanatory or predictive power of the theory. This was essentially proven by Kim in his classic treatment of supervenience (Kim 1998). Such a theory leaves it as a massive cosmic coincidence that fire hurts and ripe fruit tastes good; that our conscious states appear to generally give “good advice” about what to do (“take your hand out of the fire!”). Consciousness is tacked on to our physical theory *ad hoc*, because we know it exists, but it does not fit naturally into a classical physical model and has no explanatory power.

But what about the quantum panprotopsychist picture we developed earlier? Isn’t consciousness in the quantum panpsychist model still epiphenomenal, in that one might imagine the same unified quantum behavior of a physical system without the unified experience occurring alongside it? No. Under the model proposed here the philosopher’s zombie is not possible. Based on our own direct experience, we have postulated a mental property of matter. In our universe, a physical system without a mental property is impossible, like a physical system without any position in space.

So no, consciousness in this picture is not epiphenomenal; because once we add the panprotopsychist postulate, the quantum theory can explain why this physical state has an associated large-scale mental experience while another physical state does not. Once we have postulated that physical states with certain specific (holistic) causal powers are associated with conscious experience, we can explain why those experiences come with the power to guide adaptive behavior and realize the advantages—in the real physical world—of having unified quantum states to compute and learn with. Now we have a principled way to understand and predict which physical states come with a large-scale conscious experience, and why those physical states confer a fitness advantage to the organisms that implement them. Conscious physical states in the quantum model do have distinct causal powers in the physical world, physical effects and computations they can carry out that incoherent states cannot.

This is the sense in which conscious physical states are not epiphenomenal in the quantum model: they have distinct causal advantages in the physical world that can explain their evolution by natural selection. I acknowledge that this proposal does not necessarily defeat the “conceivability argument” (Chalmers 2013), in that zombies may remain “conceivable” under Orch OR. I submit that the important sense of the EP is that we cannot explain the evolution of consciousness, whereas the conceivability issue is a less fundamental problem originating from our incomplete knowledge. For science, the important thing is that we can understand how organisms could evolve these physical states that correspond to useful information about the external and internal environment, and why it would be these ones that come with consciousness. In this sense, from an evolutionary perspective, the quantum consciousness proposal does solve the EP.

If we allow ourselves to add a postulate regarding the relation between the physical Orch OR process and consciousness to our physical theory, the psychophysical bridging law, then we will have conceptually solved the EP, the CP, and the HP of consciousness (Chalmers 1997). In Orch OR, this bridging law is given by the identification of moments of Orch OR with moments of conscious experience expressing matching physical and mental “aspects.” Note again that this powerful conceptual step is “not an option” for a classical model. Solving the CP (or BP) is thus the primary motivation for the quantum consciousness proposal because solving the CP this way also solves the EP.

## Reason, understanding, creativity, and the psychological arrow of time

So what about Penrose’s argument that consciousness is noncomputable, or nonalgorithmic, based on Gödel’s theorem (Penrose 1989, 1994)? The Gödel–Lucas–Penrose argument is that a human can recognize the truth of a statement constructed to be unprovable by any given formal system, so human understanding cannot be reduced to an algorithm. Searle’s Chinese Room provides an argument for a similar conclusion that understanding cannot be reduced to an algorithm, but it has also been contentious (Cole 2024). We can dissociate this part of the Orch OR proposal from the broader hypothesis that the brain substrate of consciousness must be the collapse of a quantum state function. Still, it appears potentially warranted given that human reason is extremely challenging to model in terms of classical neural networks (Pulvermuller et al. 2021). Moreover, this aspect of the theory provides a basis for Whitehead’s ontology founded on a notion of “creativity” or “freedom” (Hameroff 2012), which is absent from Russell’s neutral monism.

And what about the connection to gravity asserted in the Orch OR model? Again, we can dissociate this aspect of the model from the general idea of a conscious quantum brain process. But quantum theory has a serious measurement problem that is not solved by decoherence (Joos and Zeh 1985, 2010, Adler 2003), which after all refers to “delocalized coherence,” not destruction of coherence (Joos and Zeh 2010). The Diosi–Penrose gravitational OR scheme provides principled, nonrandom collapse events (Diosi 1987, 1989, Penrose 1996, 2014). If that particular theory of nonrandom OR is not supported by future experiments, there are others we can consider (Mavromatos and Nanopoulos 1997, Adler and Bassi 2016). If one accepts the reality of wave function collapse (i.e. objective state vector reduction, OR), this provides an explanation for the physical arrow of time. Although it is widely believed that the second law of thermodynamics explains the arrow of time, this statistical explanation is incoherent without some irreversibility in the fundamental physical dynamics (Penrose 2009, Kastner 2017). Identifying the orchestrated reduction events with conscious moments (as in Orch OR) explains the conscious experience of irreversible time, which is absent from a deterministic classical model. If one imagines that an arrow of time may emerge statistically despite deterministic time-symmetric fundamental dynamics, one may dismiss this putative benefit. But no other model accounts for the psychological arrow of time because they are all supposed to be based on time-symmetric (classical or quantum) physics in the brain. Thus, this advantage of Orch OR should not be dismissed lightly, since it explains another essential characteristic of our conscious experience, which no other model accounts for.

## Conclusion

The quantum consciousness hypothesis is often derided as “two mysteries explaining each other.” Consciousness is mysterious, and quantum mechanics is mysterious, so in the stereotype the starry-eyed neuroquantophile simply sets the two mysteries equal to each other and declares them both solved. In this connection, the failure of classical physics to allow for unified states like our conscious experiences does not prove that quantum physics has the answer—except that quantum physics has exactly the relevant property that is missing from classical physics: irreducible causally efficacious holism, ontological unity, objectively integrated information.

With the theoretical HP solved at the conceptual level, the field of consciousness science may now face a psychological HP because developing the quantum approach to a fundamental naturalistic account of consciousness will require physicists to learn about biology and biologists to learn about quantum theory.
